# Genetic signature of Last Glacial Maximum regional refugia in a circum-Antarctic sea spider

**DOI:** 10.1098/rsos.170615

**Published:** 2017-10-18

**Authors:** Anna Soler-Membrives, Katrin Linse, Karen J. Miller, Claudia P. Arango

**Affiliations:** 1 Unitat de Zoologia, Universitat Autònoma de Barcelona, 08193 Bellaterra, Barcelona, Spain; 2 Biodiversity and Geosciences Program, Queensland Museum, PO Box 3300, South Brisbane, Queensland 4101, Australia; 3 British Antarctic Survey, Natural Environmental Research Council, High Cross, Madingley Road, Cambridge CB30ET, UK; 4 Australian Institute of Marine Science, Indian Ocean Marine Research Centre Fairway, cnr Service Road 4, Crawley, Western Australia 6009, Australia

**Keywords:** *Nymphon australe*, phylogeography, Southern Ocean, DNA barcoding, population structure

## Abstract

The evolutionary history of Antarctic organisms is becoming increasingly important to understand and manage population trajectories under rapid environmental change. The Antarctic sea spider *Nymphon australe*, with an apparently large population size compared with other sea spider species, is an ideal target to look for molecular signatures of past climatic events. We analysed mitochondrial DNA of specimens collected from the Antarctic continent and two Antarctic islands (AI) to infer past population processes and understand current genetic structure. Demographic history analyses suggest populations survived in refugia during the Last Glacial Maximum. The high genetic diversity found in the Antarctic Peninsula and East Antarctic (EA) seems related to multiple demographic contraction–expansion events associated with deep-sea refugia, while the low genetic diversity in the Weddell Sea points to a more recent expansion from a shelf refugium. We suggest the genetic structure of *N. australe* from AI reflects recent colonization from the continent. At a local level, EA populations reveal generally low genetic differentiation, geographically and bathymetrically, suggesting limited restrictions to dispersal. Results highlight regional differences in demographic histories and how these relate to the variation in intensity of glaciation–deglaciation events around Antarctica, critical for the study of local evolutionary processes. These are valuable data for understanding the remarkable success of Antarctic pycnogonids, and how environmental changes have shaped the evolution and diversification of Southern Ocean benthic biodiversity.

## 1. Introduction

The Southern Ocean (SO) marine biota is unique in terms of ecology, phylogeography and diversity [1] as a consequence of the long isolation, the unique geological and climatological history, and recurrent glacial cycles. There is evidence that ice sheets covered the Antarctic continental shelves during the glaciation periods [2] and until recently, models of the ice sheet cover and extent during the Last Glacial Maximum (LGM) (approx. 70–10 ka) predicted ice sheet advances to the shelf break of most of the Antarctic continent [3]. The general thought then has been that species presently found on the Antarctic shelf (including terrestrial species) had to recolonize the Antarctic continental shelf and the ice-free low altitude terrestrial surfaces from elsewhere, e.g. the deep sea or the southern margins of other continents [4,5]. However, recent biogeographic and molecular genetic analyses on Antarctic marine and terrestrial taxa give strong evidence that both shelf and terrestrial taxa could have also survived in ice-free habitats through glacial periods [6–10]. Also, the most recent reconstructions of the Antarctic Ice sheet during the LGM suggest that it did not reach the shelf edge all around Antarctica and that the spatial pattern of deglaciation was highly variable, especially on inner shelves [11], enabling the presence of shelf refugia. This variability in the nature of ice extent and the way in which it retreated probably had differential effects on the recolonization and expansion of marine populations, resulting in different evolutionary trajectories around Antarctica. The multi-national, SO-wide expeditions linked to the Census of Antarctic Marine Life (CAML) enhanced the collection, sharing and barcoding of specimens [12], enabling detailed phylogeographic analyses on selected taxa to study their relatively recent evolutionary history [8,9,13–17]. These datasets are now contributing to understanding how populations around Antarctica survived through past environmental change.

One of the key features of the Antarctic benthos is the relatively high abundance and diversity of Pycnogonida (sea spiders); more than 20% of the global species diversity of this ancient, cosmopolitan Class distantly related to Chelicerata [18,19] is found in the SO. With high rates of endemism (70%) and an apparent lack of biogeographic subregions [20,21], the SO pycnogonid diversity suggests that species have not only survived past climatic events but successfully dispersed and diversified; however, mechanisms and patterns of dispersal and diversification are yet unknown.

In general, pycnogonids are known as brooders with no planktonic dispersal stage as the fertilized eggs and sometimes post-larval stages are usually attached to the male [22]. This condition has led to assumptions of limited dispersal, high speciation rates and the likely presence of cryptic species [23]. Recent studies have investigated the validity of apparently circumpolar, eurybathic and abundant SO pycnogonid species using the partial cytochrome *c* oxidase subunit I (COI) barcoding marker for: *Pallenopsis* spp. [15,24], *Austropallene cornigera* (Möbius, 1902) [25], *Colossendeis* spp. [16,26], and *Nymphon australe*, the first species studied at the population level using COI [13,27].

*Nymphon australe* Hodgson 1902 is the most abundant and frequently collected SO species with wide bathymetric (8–4136 m depth) and geographical ranges. It has been recorded from around the Antarctic continent, the southern tips of South America, South Africa and New Zealand, although New Zealand representatives are considered a subspecies *N. australe caecum* Gordon, 1944 [20,28]; in general, non-SO records are yet to be validated with genetic data. Based on morphological characteristics of SO specimens, *N. australe* is a single widespread species with intraspecific variation of some characters sometimes overlapping interspecific differentiation [13]. Initial molecular studies examining the species validity of *N. australe*, based on 57 specimens from the Antarctic Peninsula (AP) [27] and later with an extended dataset including 74 specimens from the Weddell Sea (WS) and East Antarctic (EA) [13], pointed to *N. australe* as a true circumpolar species with distinct genetic differentiation among individuals from different localities and regions.

The present study aimed to: (i) investigate the genetic differentiation and demographic histories of *N. australe* populations around Antarctica to understand the wide geographical distribution proposed for the species, and (ii) relate the genetic structure observed with historic events including refugia survival during LGM and population expansion during deglaciations. We examined the genetic structure of the species at a range of spatial (1000s km to 100s km) and bathymetric scales taking advantage of: (i) large geographical coverage of the dataset, one of the largest for benthic SO invertebrates; (ii) wide bathymetric range with individuals collected from 25 to 1260 m depth; and (iii) the unusual and relatively large number of individuals collected at several locations in the EA.

## 2. Material and methods

### 2.1. Sampling and taxonomy

**Figure 1.**
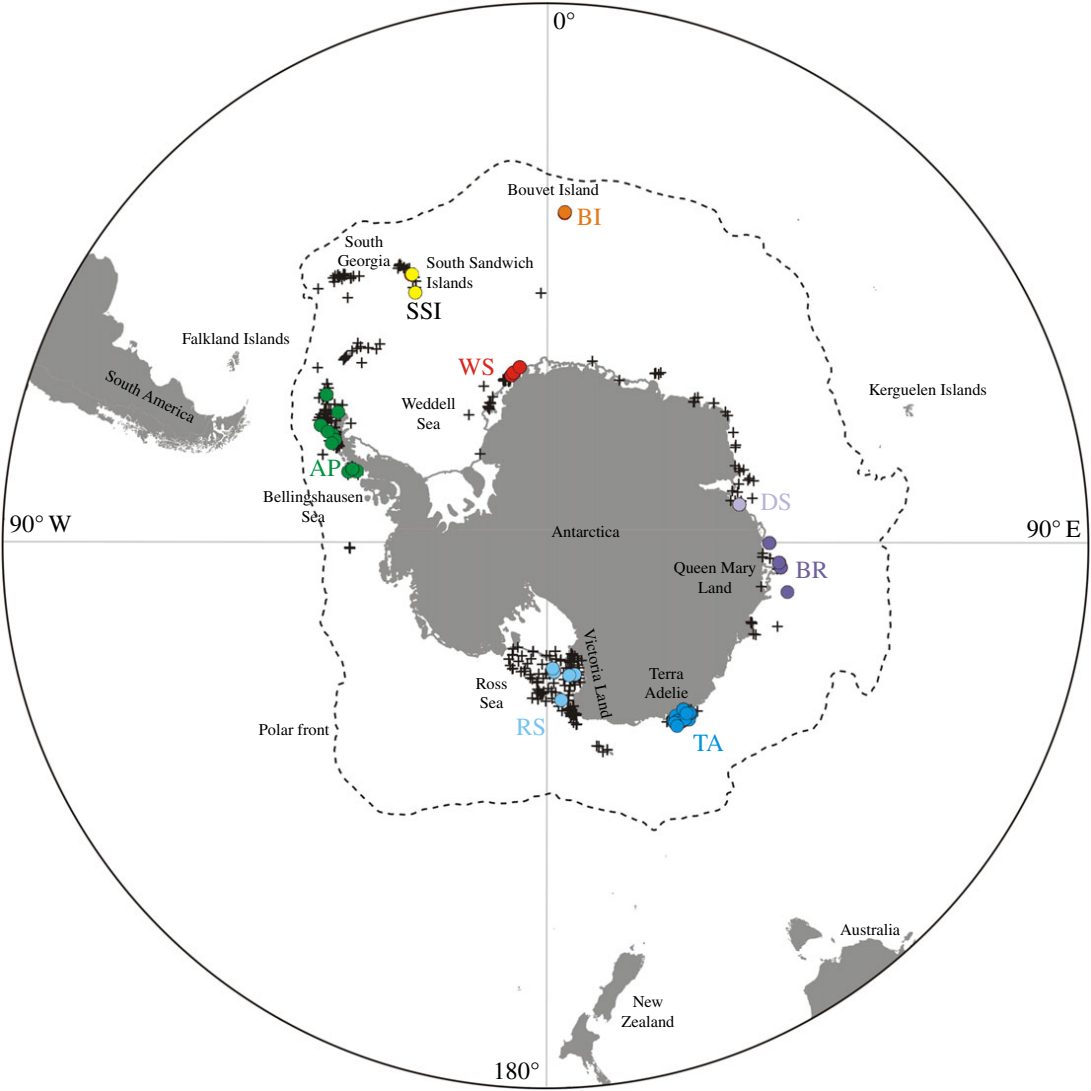
Records of *N. australe* from the Southern Ocean. Coloured circles represent location of the samples used in this study. Crosses indicate historical records of *N. australe* showing known distribution of the species. TA, Terre Adélie; RS, Ross Sea; DS, Davis Station; BR, Bruce Rise; AP, Antarctic Peninsula; WS, Weddell Sea; SSI, South Sandwich Islands; BI, Bouvet Island.

The present dataset is based on material from institutional projects and collections, and from international collaborative efforts (under CAML) aiming to collect, determine and curate well-preserved SO benthic fauna [29]. Expeditions details and voucher information are in the electronic supplementary material, S1 and S2. The dataset consists of 364 *N. australe* specimens collected by a variety of sampling means from diving to trawling at 69 sites in the SO between 2007 and 2011 (figure 1; details of localities in the electronic supplementary material, S1). Pycnogonids collected during expeditions were preserved in 96% ethanol and posted to the Queensland Museum (QM) for morphological determination by CPA and further analyses. Molecular identification was based on COI divergences for *Nymphon* species as in Mahon *et al*. [27] and Arango *et al*. [13]. Sequences from previous studies are included in the current dataset (haplotypes GenBank accession numbers in the electronic supplementary material, S3).

### 2.2. Mitochondrial DNA sequencing and data analysis

Most of the samples were submitted to the Canadian Centre for DNA Barcoding as part of the barcoding agreement between CAML [11] and the Canadian Centre for DNA Barcoding; DNA extractions and COI sequencing were performed under their standard procedures [30]. Additional sequences were obtained at the QM following protocols in Arango *et al*. [13]. Other genetic markers such as fragments of the rRNA 16S (463 bp, 57 individuals) and the rRNA ITS including ITS1, 5.8S, ITS2 and small parts of the 18S and 28S regions (890 bp, 15 individuals) were attempted for a multi-marker approach; however, the fragment of rRNA 16S showed no sequence variation [13] and the rRNA ITS fragment attempted for 15 samples from the WS and East Antarctica sequenced as in Arango & Brenneis [31] showed very little divergence (*p*-distance ≤ 1%), discouraging the use of this ITS fragment for within-species level analyses in *N. australe.* The use of microsatellites to complement this COI dataset was also attempted; however, the developed markers were too conserved. Four markers were monomorphic for all analysed samples with half of them showing no polymorphism even across species boundaries [32].

Uncorrected pairwise distances among the COI sequences were calculated with 1000 bootstrap replicates under a J–K model in MEGA v. 7 [33]. *Nymphon mendosum* (Hodgson, 1907), an Antarctic species closely related to *N. australe* [13], was included as a reference of interspecific distance. The overall *p*-distance mean among *N. australe* sequences was 0.01 while between *N. australe* and *N. mendosum* was 0.08. A haplotype parsimony network was constructed using TCS 1.21 [34] with 95% connection limit. A haplotype matrix was analysed under a Bayesian inference approach (BI) in MrBayes 3.1.2 [35] using MrModelTest v.2 [36]. Based on the Akaike information criterion [37], the HKY + I + K was chosen. Two simultaneous sets of one chain were run for subsequent sets of numbers of generations sampling trees every 100 generations (ngen = 8 million; nruns = 2, nchain = 1; samplefreq = 100). After this number of generations, the standard deviation of split frequencies had reached 0.02 and the potential scale reduction factor was 1.00 for all parameters suggesting convergence had been reached.

### 2.3. Genetic diversity and population structure

Based on the sampling localities, COI sequences were grouped in one of four main SO geographical regions: Antarctic Peninsula (AP), East Antarctic (EA), Weddell Sea (WS) and Antarctic Islands (AI). Furthermore, the EA was divided into four subregions: Terre Adélie (TA), Ross Sea (RS), Davis Station (DS) and Bruce Rise (BR). Within the AI, there were two subregions: Bouvet Island (BI) and South Sandwich Islands (SSI) (electronic supplementary material, S1, S2; figure 1). Genetic diversity of *N. australe* was measured for each SO region and for the subregions within the EA using DNASP v. 5.00.7 [38]. Departures from values expected under panmixia (i.e. *F*_ST_ = 0) among SO regions and within the EA, and the corrected *p*-values for population differentiation among pairs of populations were tested with Arlequin v. 3.0.1 [39] with 10 000 permutations of the data. The partitioning of genetic variation among subregions within the EA was determined using analysis of molecular variance (AMOVA) based on 2000 permutations.

To infer the spatial genetic structure of *N. australe,* the number and the composition of panmictic groups as well as the spatial boundaries among them were estimated using a Bayesian model computed with Geneland v. 2.0.0 [40] in the R environment (R, v. 3.2.3 [41]). The software implements a Markov chain Monte Carlo (MCMC) procedure to determine the best clustering of samples based on genetic and geographical information. Geographical information has been taken into account at the Bayesian prior level, so that clusters corresponding to spatially structured groups are considered to be more likely than clusters that are randomly distributed in space. Five million MCMC iterations sampled each 1000 steps with a 50 000 burn-in period, and a maximum number of clusters *K* = 10 were run to estimate the model parameters and posterior probabilities of group membership (*P*).

### 2.4. Demographic analysis

To detect demographic changes such as population expansion or contraction in *N. australe* at various spatial and temporal scales related to glacial refugia [42], the number of private haplotypes (PH, those endemic to subregions or regions) and the proportion of private haplotypes for each region (number of unique haplotypes/total number of haplotypes) was calculated. Tests for neutrality (Tajima's *D* and Fu's *F_S_*) were run in DnaSP to estimate deviations from the mutation-drift equilibrium and infer past population processes. The observed distribution of pairwise differences between sequences (mismatch distribution analysis) was examined in Arlequin under a model of population expansion and the resulting shape of the distribution was compared to a simulated dataset under a spatial expansion model [43,44] and a sudden population expansion. The overall validity of the estimated demographic model was evaluated by two different goodness-of-fit tests, tests of raggedness index (RAG) and the sum of squared differences (SSD) [45]. Significance of RAG and SSD were assessed by parametric bootstraps (10 000 replicates).

We used the coalescent-based method implemented in Fluctuate 1.4 [46] to estimate the exponential rate of population growth/decline relative to the neutral mutation rate (*g*) and the *θ* parameter (the effective population size scaled by the mutation rate, i.e. *N*_e_*μ*), the initial value of *θ* was estimated using the approach of Watterson [47]. All runs employed the following strategy: 1000 short chains with 200 generations and two long chains of 400 000 steps. Sampling increment was 20 for short and long chains. The transition/transversion rate was set to 3.99, determined by MEGA5 under the Tamura–Nei model. Runs were repeated five times to ensure consistency of estimates. Based on the significant genetic differentiation found among Antarctic regions, genetic panmixia cannot be assumed to analyse the demographic history of the whole Antarctic dataset; instead, each of the four regions is separately analysed. For the same reason, demographic analyses within EA were also run after excluding the significantly different DS samples (figure 1).

Molecular clock estimates were used to approximately date the timing of population expansions as in Rogers [48], calculating (*T*) for the analysed COI fragment based on *T* = *τ*/(2*µk*), where *τ* is the expansion time estimator, *µ* the mutation rate and *k* the sequence length [49]. The demographic expansion parameters of initial and final effective population sizes were estimated following *θ*_0_ = 2*N*_0_*u* (before population growth/decline) and *θ*_1_ = 2*N*_1_*u* (after the population growth/decline). Here, we apply a 10-fold correction when estimating *N. australe* population expansion times, as it has been suggested that the short-term mutation rate is 10-fold higher than long-term substitution rates [50,51].

## 3. Results

### 3.1. Genetic diversity and geographical structure

**Figure 2.**
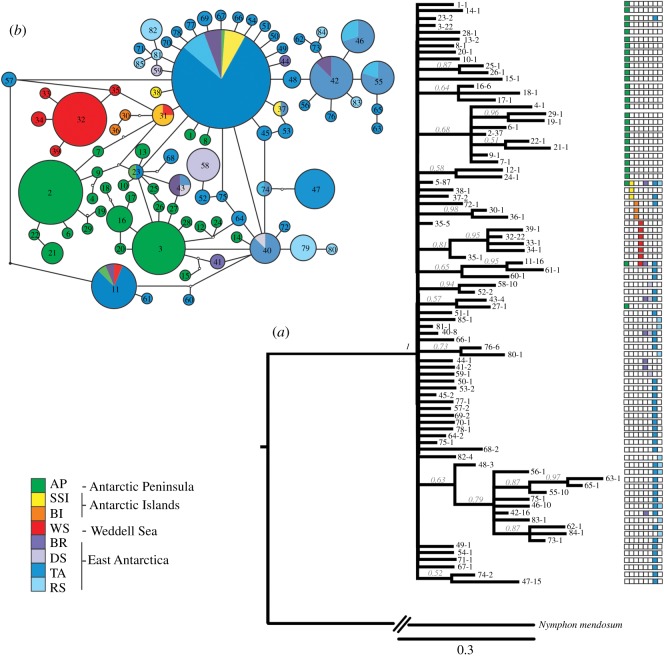
(*a*) Phylogenetic analysis of the 85 COI haplotypes. (*b*) Mitochondrial COI haplotype network of *N. australe* showing the haplotypes identified throughout the Antarctic locations. Haplotypes are coloured by region and the size of their circle is proportional to its frequency in the whole sampling effort. White small circles represent haplotypes that have not been collected but should exist.

The final COI alignment of 364 sequences of *N. australe* had a length of 554 bp and represented 85 unique haplotypes (see the electronic supplementary material, S2 and S3) of which 10 were common haplotypes (represented by 10–87 individuals), while the majority were uncommon haplotypes with only one or two representatives (figure 2). We detected intermediate levels of genetic polymorphism, 59 nucleotides were variable and 35 were parsimony informative*.* The overall mean distance among sequences was 0.007 (range 0–0.02).

The genetic diversity varied across the SO regions. The number of haplotypes (*k*) was lowest in AI (*k *= 6) and highest in EA (*k *= 50), although this was probably related to the different sampling effort in each locality given that haplotype diversity was similar in these two locations (see the electronic supplementary material, S1 and S3). Genetic variation was low in the WS, which had around half the haplotypic diversity of the other localities, as well as the lowest average number of nucleotide differences (*Π*) and mean nucleotide diversity (*π*). The number and the proportion of private haplotypes were lowest in AI, although this was the region with the smallest sample size (table 1).

**Table 1.** Genetic diversity indices and neutrality tests of *N. australe* COI sequences from each Antarctic region, the entire Antarctic dataset, and areas within the EA region. (*k*, number of haplotypes; PH, number of private haplotypes; H, haplotype diversity; *S*, number of polymorphic sites; *Π*, average number of nucleotide difference; *π*, nucleotide diversity.)

	*n*	*k*	PH	PH/*k*	*H*	*S*	*Π*	*π*
locality								
East Antarctica	227	50	46	0.920	0.857	39	2.748	0.00496
Antarctic Peninsula	95	29	26	0.897	0.794	29	2.981	0.00538
Antarctic Islands	13	6	3	0.500	0.769	5	1.526	0.00275
Weddell Sea	29	7	5	0.714	0.429	8	0.739	0.00133
all	364	85	—	—	0.918	59	3.642	0.00657
East Antarctic								
Davis Station	13	4	2	0.500	0.423	7	1.769	0.00319
Bruce Rice	19	7	2	0.286	0.854	9	1.988	0.00359
Terre Adélie (CEAMARC)	154	39	24	0.615	0.815	32	2.671	0.00482
Shallow Terre Adélie (REVOLTA)	13	7	0	0.000	0.795	6	1.513	0.00273
Terre Adélie (CEA + REV)	167	39	30	0.769	0.812	32	2.592	0.00468
Ross Sea	28	11	7	0.636	0.878	13	2.698	0.00487
Antarctic Islands								
Bouvet Island	5	3	2	0.667	0.700	3	1.6	0.00289
South Sandwich Islands	8	3	1	0.333	0.464	2	0.5	0.00090

There was significant genetic differentiation among the four regions sampled around Antarctica (AMOVA, *F*_ST_ = 0.424, *p* < 0.001). COI pairwise *F*_ST_ relationships and significance comparisons supported the structuring of SO regions (table 2), with relatively high and significant *F*_ST_ values among all localities. WS samples were the most distinct from all other regions (*F*_ST_ between 0.51 and 0.68), while *F*_ST_ values between EA and AI samples were the lowest, but still significantly greater than zero (*F*_ST_ = 0.10).

**Table 2.** Pairwise COI *F*_ST_ values among the distinct Antarctic regions analysed in *N. australe*. (All values were significant (*p* < 0.05).)

	East Antarctica	Antarctic Peninsula	Weddell Sea	Antarctic Islands
East Antarctica	0			
Antarctic Peninsula	0.38208	0		
Weddell Sea	0.51120	0.58352	0	
Antarctic Islands	0.10015	0.35711	0.68291	0

**Figure 3.**
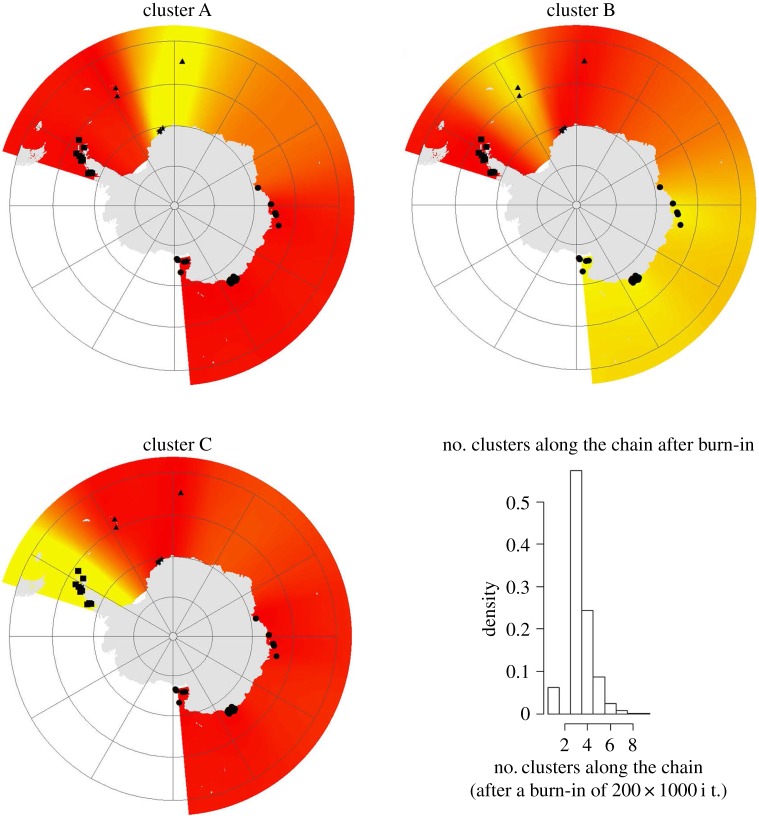
Spatial representation and relative location of sampled *N. australe* populations. Number of clusters predicted based on the Bayesian clustering algorithm shown as per figure on bottom right (*K* = 3; Geneland output). Cluster A, Weddell Sea and Bouvet Island; cluster B, South Sandwich Islands and East Antarctica; cluster C, Antarctic Peninsula. Based on this spatial output, darker and lighter shading are proportional to posterior probabilities of membership clusters, with lighter (yellow) areas showing the highest probabilities of clusters. Number of clusters predicted with the Bayesian clustering algorithm.

A Bayesian phylogenetic analysis did not show segregated, well-supported clades related to region; however, the COI haplotype network coupled with the Bayesian consensus while supporting *N. australe* as a single species with a circum-polar distribution, also showed clear regional differences in the distribution of haplotypes (figure 2*a*). Most haplotypes were found only within a single region except for the AI where a large proportion of haplotypes were found elsewhere. Furthermore, the haplotype network had a predominant star-like pattern at the regional level, particularly for EA (figure 2*b*). Bayesian models computed with Geneland detected three main clusters (*K* = 3, figure 3): cluster A included individuals from the WS and BI (figure 3*a*), cluster B grouped all EA individuals with the SSI (figure 3*b*), and cluster C included all AP individuals (figure 3*c*). Values of cluster membership were approximately *P* = 0.65 for each cluster locality.

**Figure 4.**
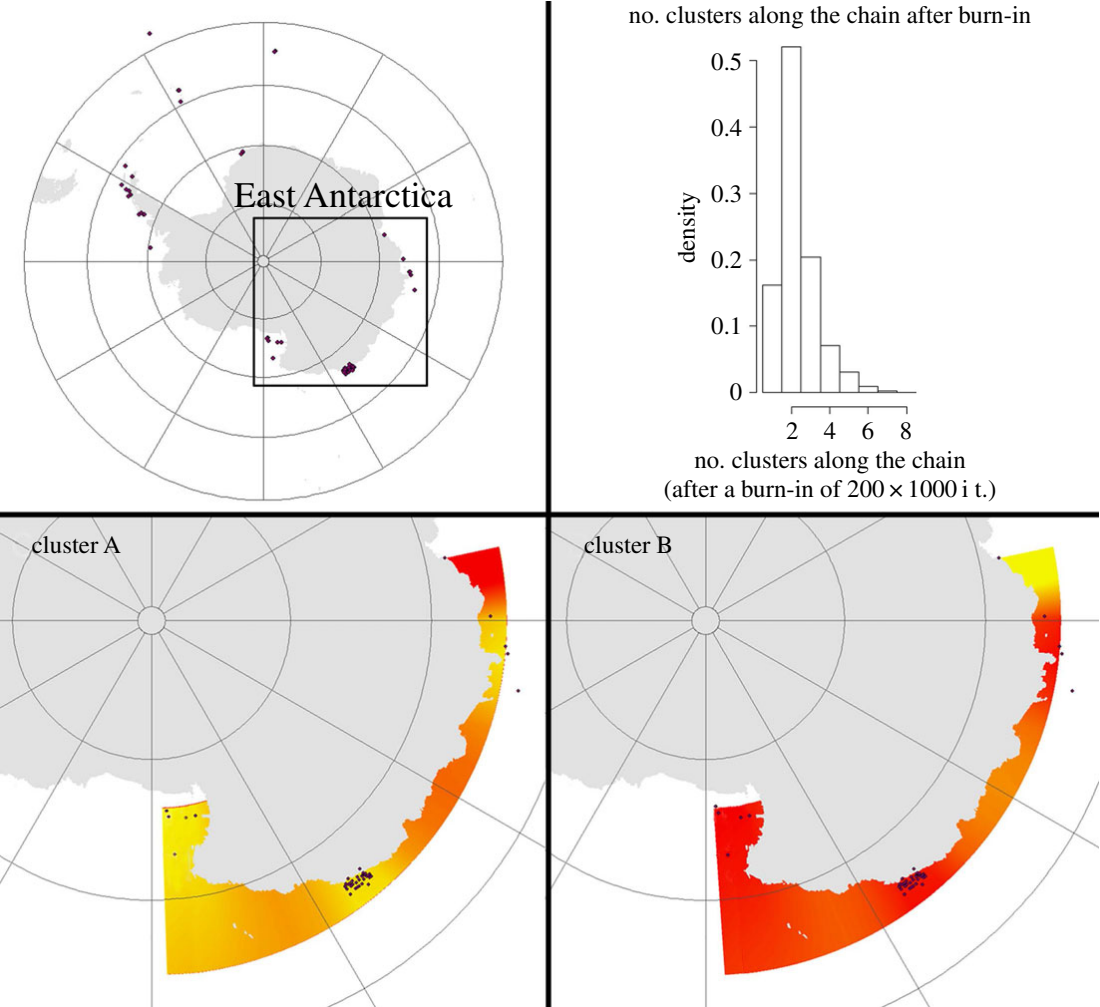
Spatial representation and relative location of sampled EA *N. australe* populations. Number of clusters predicted based on the Bayesian clustering algorithm as per figure on top right (*K* = 2; Geneland output). Cluster A, Bruce Rise, Terre Adélie, Ross Sea; cluster B, Australian Davis Station. Darker and lighter shading are proportional to posterior probabilities of membership clusters, with lighter (yellow) areas showing the highest probabilities of clusters.

AMOVA and *F*_ST_ results within the AP and WS indicated low differentiation within regions, over 90% of the variation was owing to the differences within populations. Within the EA region, there was significant genetic differentiation among the five subregions sampled (AMOVA, *F*_ST_ = 0.238, *p* < 0.001), although this was largely a result of the large and significant differences between DS samples and those from all other subregions (table 3). We also detected low but significant differences between the RS and other EA subregions (table 3), although the TA and BR populations were not differentiated (among these populations *F*_ST_ < 0.025). Bayesian cluster analysis revealed two clusters (*K* = 2; figure 4), one included individuals collected in the vicinity of the Australian DS (cluster B, figure 4), and the other one grouped all other EA populations (BR, TA and RS) (cluster A, figure 4). Values of cluster membership were high for all localities (*p* > 0.90). Samples from DS were all collected in about 25 m depth, whereas most other EA samples were collected deeper than 200 m. A subset of the TA samples collected by the REVOLTA program from approximately 40 m depth showed no evidence of significant genetic differentiation from deeper TA samples (table 3), suggesting that segregation of the DS vicinity populations could be related more to geographical isolation than mere bathymetric differences.

**Table 3.** Pairwise COI *F*_ST_ values among the distinct areas within the EA region analysed in *N. australe*. (All significant values (*p* < 0.05) are shown in italics.)

	DS	BR	TA	shallow TA	RS
DS	*0*				
BR	*0*.*49287*	*0*			
TA	*0*.*40637*	0.02080	*0*		
shallow TA	*0*.*52101*	0.02232	0.01230	*0*	
RS	*0*.*46385*	*0.06553*	*0.05120*	*0.12237*	*0*

Genetic differentiation within AP was significant owing to differences between northeastern and southernmost populations of the AP (*F*_ST_ = 0.08, *p* < 0.05). The AI populations (BI and SSI) were also significantly different (*F*_ST_ = 0.51, *p* < 0.05), while the WS did not show significant differences (*F*_ST_ = 0.03, *p* > 0.05) between the easternmost and westernmost populations. (Within-regions AMOVA and *F*_ST_ results in the electronic supplementary material, S4.)

### 3.2. Demography

The significantly negative values of *D* and *F_S_* and unimodal mismatch distributions obtained for the whole dataset (Tajima's *D* = −1.842, *p* < 0.05; Fu's *F_S_* = −95.972, *p* < 0.001) seem to indicate that *N. australe* populations have gone through relatively recent expansions. We obtained a similar pattern in separate analyses of the WS, EA and AP data. Either an L-shaped or a unimodal distribution of pairwise differences was obtained for each region, supporting the existence of a demographic expansion in each region; this pattern was corroborated by the negative and significant Fu's *F_S_* test values and the non-significant goodness-of-fit tests. Tajima's *D* was not significant (*p* > 0.05) for AP and EA, suggesting there has not been historical reduction in effective population size in these regions (table 4; electronic supplementary material, S5).

**Table 4.** Historical demography parameters inferred for the entire COI dataset of *N. australe* as well as for each region: East Antarctica (EA), Antarctic Peninsula (AP), the Antarctic Islands (AI) and WS. For the mismatch analysis, estimates simulated under a spatial expansion (spa.exp.) and a demographic expansion (dem.exp.) are listed. (When estimating the population expansion time, the 10-fold correction is applied. s.d. indicates the standard deviation and CI confidence interval ranges. Asterisks (*/**) indicate a significance level of *p* < 0.05/0.01, respectively.)

	EA	EA without DS	AP	AI	WS
neutrality tests					
Tajima's *D*	−1.728		−1.502	−0.779	−1.951*
Fu's *F_S_*	−49.372**		−17.764**	−1.874	−3.961*
mismatch analysis					
tau_spa.exp._ (5–95% CI)	3.107 (1.568–4.469)	2.727 (1.424–4.352)	3.840 (2.066–6.327)	0.688 (0.569–4.733)	0.194 (0.000–3.156)
theta 0_spa.exp._ (5–95% CI)	0.001 (0.001–1.538)	0.093(0.0001–1.462)	0.235 (0.001–1.542)	0.923 (0.001–1.966)	0.438 (0.001–0.449)
theta 1_spa.exp._ (5–95% CI)	8.897 (3.686–61.531)	16.583 (3.237–138.516)	4.139 (1.607–15.371)	11833.006 (0.542–inf)	52.705 (0.000–inf)
tau_dem.exp._ (5–95% CI)	3.432 (1.865–5.494)	3.019 (1.620–4.851)	4.691 (2.182–8.004)	1.312 (1.266–3.273)	0.943 (0.000–3.305)
theta 0_dem.exp._ (5–95% CI)	0.005 (0.000–1.021)	0.003 (0.000–1.026)	0.002 (0.000–1.020)	0.018 (0.000–0.666)	0.000 (0.000–0.113)
theta 1_dem.exp._ (5–95% CI)	9.189 (5.312–87.626)	29.393 (4.477–120.827)	6.406 (4.363–43.281)	3483.156 (5.752–162.539)	0.947 (0.591–447.829)
SSD (*p* value)	0.004 (0.505)	0.009 (0.462)	0.032 (0.130)	0.021 (0.090)	0.004 (0.753)
RAG index (*p* value)	0.02 (0.675)	0.040 (0.498)	0.073 (0.123)	0.111 (0.077)	0.136 (0.506)
demographic parameters					
*g* (s.d.)	2187.139 (169.970)	2033.550 (142.738)	3534.656 (210.318)	6333.006 (480.841)	8151.244 (403.155)
*θ* (s.d.)	0.092 (0.006)	0.081 (0.007)	0.193 (0.032)	0.101 (0.037)	0.071 (0.012)
time of expansion (in KY bp)	154.857	136.236	211.706	59.228	42.571

The *N. australe* samples from the islands (AI) showed non-significant neutrality tests, rejecting the population expansion model (table 4). Although the goodness-of-fit tests indicated that the model of rapid population expansion cannot be rejected, the calculated probability was very close to the significance level (*p* = 0.09 and 0.07 for SSD and RAG, respectively) (table 4). However, the unimodal mismatch distribution of pairwise differences suggested a recent demographic expansion (table 4; electronic supplementary material, S5). The estimates of change in population size relative to the mutation rate were consistent with previous analyses and ensured convergence on the correct parameter estimates for each region; replicate runs with alternate random seeds produced comparable results. Results of the coalescence analysis showed positive values providing evidence of different degrees of population expansion at each of the four regions, ranging from *g* = 2187 (s.d. = 170.970) in EA to *g* = 8151 (s.d. = 403.155) in WS (table 4).

**Figure 5.**
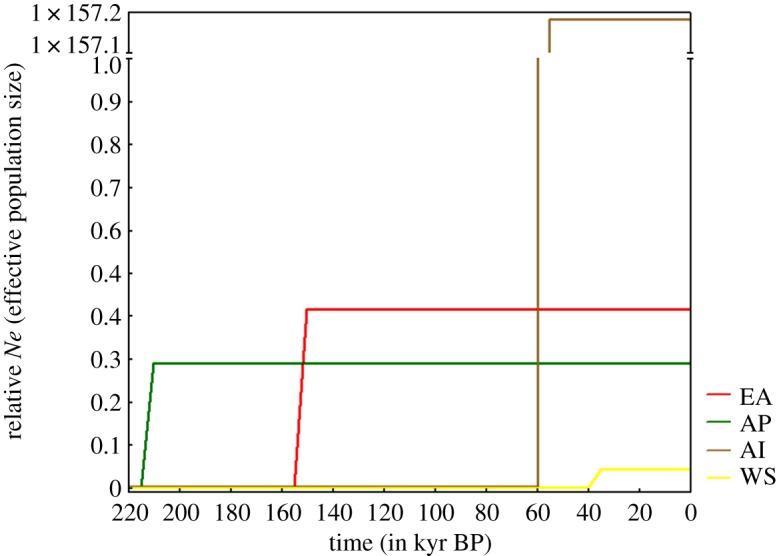
Demographic expansion time estimates for *N. australe* based on the COI gene fragment.

Sudden growth model analyses detected a population expansion at about 15 thousand years before present (kyr BP) at EA (13 kyr BP at EA excluding DS from the analysis) and an older expansion at the AP (21.2 kyr BP). These estimates (obtained after applying the 10-fold correction) are in agreement with the high genetic diversity calculated for each of the two regions. On the other hand, WS and AI populations with lower values of haplotype and nucleotide diversity seem to have gone through more recent population expansions around 4.3 and 5.9 kyr BP, respectively (figure 5).

## 4. Discussion

The circum-Antarctic sea spider, *N. australe* is comprised of regionally distinct populations that appear to have undergone recent population expansion. Here, we show: (i) further evidence to confirm that *N. australe*, a benthic brooder, is indeed circum-Antarctic; (ii) that the AP and EA populations are genetically more diverse suggesting multiple demographic contraction–expansion events possibly associated with deep-sea refugia, while the low genetic diversity at the WS points to a more recent expansion possibly of shelf ice-free refugia; (iii) *N. australe* seems to have undergone demographic expansions during the mid-Pleistocene (15–21.2 kyr BP), suggesting multiple LGM refugia; (iv) support for population expansion in the AI associated with colonization from the continent, and (v) no genetic boundaries detected in East Antarctica, except for the clear segregation of samples from the vicinity of DS, an area particularly isolated from the more open habitats sampled off TA.

### 4.1. *Nymphon australe*, a circum-Antarctic species

Our study supports previous work proposing *N. australe* as a circum-Antarctic species [13]. Here, we found relatively high genetic homogeneity in COI among the *N. australe* individuals from across Antarctica that together formed a single haplotype network precluding any suggestion of cryptic speciation (either geographical or bathymetric) within the species. Although the use of a single mitochondrial marker is often considered limiting in the context of detecting recent speciation and inferring spatial genetic structure [9,52], our efforts to incorporate additional genetic markers were futile as there was low or no intraspecific sequence divergence, even for nuclear markers that should be highly variable, e.g. microsatellites [32] and ITS which has been successful in segregating within-species clades in other pycnogonid species [16,31]. Thus, COI appears to be the marker of choice for interrogating genetic patterns in *N. australe*.

Pycnogonids in general are thought to be poor dispersers; for a benthic invertebrate with no pelagic stages, slow-moving, and with fertilized eggs and post-embryonic stages remaining attached to the father for some variable time, a circum-Antarctic distribution seems unusual. In other Antarctic pycnogonids studied, such wide distributions have been challenged, and restricted gene flow eventually leading to cryptic speciation has been proposed (e.g. *Colossendeis megalonyx* Hoek, 1881 [26,53]; *Pallenopsis patagonica* (Hoek, 1881) [15,24]); similar patterns are evident for many other Antarctic invertebrates too (e.g. Isopoda [54–57]; Amphipoda [58,59]; Ostracoda [60], Nudibranchia [61]; Crinoidea [52,62]). By contrast, data for Antarctic taxa with pelagic stages do not give evidence of geographical genetic structure and tend to reflect a circum-Antarctic distribution [9,63,64]. The absence of allopatric speciation in *N. australe* is thus surprising, and raises questions on how to reconcile a wide distribution, more characteristic of a species with pelagic dispersal, with the life-history traits of the species (benthic, crawler, late post-larval instars carried by the male [65]). The roles that environmental (e.g. ocean currents and ice movement) and ecological (e.g. hosts associations, drifting substrates, parasitism) factors may play in the maintenance of a circum-Antarctic species and Antarctic pycnogonid populations in general are yet to be studied. On the other hand, the clear genetic differentiation among Antarctic regions (*F*_ST_, table 2; haplotype network, figure 2) may well be seen as evidence of premature stages of speciation [13]. Unfortunately, no fossil records exist that help understanding the species history; assuming populations were established posterior to the LGM, it should be considered whether there have been sufficient generations for speciation to occur.

### 4.2. High genetic diversity and population structure of *N. australe*

The genetic diversity estimated for *N. australe* (*H* = 0.918; *π* = 0.00657) is higher than that for other invertebrates distributed throughout Antarctica including echinoderms [66] and arthropods [60] in which either distinct morphospecies are detected, or genetic homogeneity is associated with the occurrence of a pelagic stage [63]. The observed genetic structure of Antarctic benthic taxa may be explained by the combination of widely dispersed and well-connected SO species, with the result of impacts of local events controlling gene flow including isolation and expansion processes. The high levels of genetic diversity in the EA and AP populations (in agreement with Mahon *et al*. [27] for the AP), higher haplotype and nucleotide diversities, high number of exclusive haplotypes and high proportions of their ancestral haplotypes might be related to region-specific conditions. On the other hand, the singular grouping of BI together with WS and SSI together with EA is rather unexpected. There is the assumption of lack of connectivity between the islands and the shelf [20] and Antarctic areas are seen as a separate zoogeographical region mainly influenced by the Antarctic Circumpolar Current (ACC). This pattern has been found in *A. cornigera*, in which distant populations on the Antarctic continental shelf (WS and TA) clustered together while the islands segregated as distinct clusters [25]. Dömel *et al*. argue that *A. cornigera* dispersal is limited by the ACC acting as major dispersal barrier, and by a relatively restricted depth range (max of 1180 m) limiting access to the deep sea. Here, the results could be seen as support for the hypothesis of connectivity between the SO islands and the continent [16,26] being enhanced by deep currents in eurybathic species such as *N. australe* (known max depth 4136 m); however, the large and significant *F*_ST_ values found indicate regions are not connected. An alternate explanation for the grouping of samples from the islands with those from the continent is that these geographical populations are isolated and are in the process of diverging, but owing to post-LGM colonization, ancestral genetic signatures might remain.

### 4.3. Past population histories differ among regions

It is understood that the differences in mutation rates across lineages may impact estimates of molecular time-scales and demographic parameters from mitochondrial sequence data [67]. Based on published datasets, a 10-fold correction of the proposed mutation rate seems to be the most accepted in the literature (see [50,68–73] among others). Applying the 10-fold correction infers a period of an order of magnitude more recent than applying no correction to the mutation rate. Here, we apply such correction to our estimates of expansion of *N. australe* populations, as corrected estimates (instead of 40–220 kyr BP with no correction applied) fit well with accepted hypotheses of benthic invertebrate refugia survival and population expansions associated with deglaciation events after the LGM about 20 kyr BP [9,25,61,71,74]. Although molecular clock estimates should be regarded with caution, and especially in Pycnogonida that still awaits dated phylogenetic hypotheses, the estimated timing of *N. australe* populations expansion (figure 5) matches that for other pycnogonid species [16,24] and other invertebrates (e.g. *Nacella concinna* [68]; *Nematocarcinus lanceopes* [63]; *Pareledone turqueti* [9]), as well as estimates of raising temperatures (end of the LGM) according to the Antarctic temperature anomaly (discussion in [75]).

There is strong evidence that, during the last glaciations, ice sheets extended to cover the continental shelves of Antarctica [76]; however, different rates of cover have been proposed for different Antarctic regions [11]. It has been suggested that the shelf benthic fauna was depleted during the LGM, but there are no sufficient data to fully understand the recolonization processes. Distinct scenarios have been proposed for explaining recolonization of the Antarctic shelf: (i) fauna found refugia *ex situ*, on the shelf of neighbouring continents or sub-AI [16,77]; (ii) fauna found refugia *in situ* on the continental slope and deeper waters of the SO [5]; and (iii) survival of fauna throughout the last glaciation *in situ* in shelf refugia [6,7,75,76,78]; a variety of taxa seems to agree with one of these scenarios ([8,16,25] and others). Allcock and Strugnell [8] reviewed studies on genetic structure of SO organisms and suggested that much of this fauna would have survived the Quaternary glaciations *in situ*. Our *N. australe* findings agree with those views, but, different hypotheses apply (2 and 3 above) depending on the region.

The demographic history inferred for the EA and AP regions seems to follow a similar pattern of *in situ* survival. All demographic analyses (table 4 and figure 5) and the considerable degree of genetic diversity observed (table 1) suggest that these regions experienced a process of population expansion with no signs of historical bottleneck (Tajima's *D* test not significantly negative). These time estimates of expansion, 15.5 kyr BP at EA (13.6 excluding the differentiated DS samples), and 21.1 kyr BP at the AP, fit with the dating of post-LGM deglaciation events in these regions (approx. 14 kyr BP in EA and approx. 19.5–16 kyr BP in AP [75,79]). It has been hypothesized that well-grounded ice sheets across the continental shelf displaced organisms from the shelf, some finding refugia on the slope or deeper waters evading population bottlenecks [7,63].

It is plausible to assume that *N. australe* populations from EA and AP are genetically diverse possibly owing to repeated colonization events from shallow to deep and deep to shallow and changing landscape owing to ice expansion and retraction, supporting the hypothesis of multiple independent glacial refugia [25]. Also, eurybathic species like *N. australe* could be expected to retain high levels of genetic diversity [16,27,63], different to taxa bathymetrically restricted to shallow waters not migrating to deeper waters and that may have suffered severe population reductions during the glacial periods diminishing their genetic diversities [68].

Contrary to those from the AP and the EA, levels of genetic diversity in *N. australe* from the WS are rather low. The strongly reduced diversity, lower estimates of theta and higher growth rates (coalescent modelling) detected in WS populations compared to EA and AP (table 4 and figure 5) agree with the hypothesis of population expansion following events that might have reduced populations to one or very few *in situ* refugia, perhaps ice-free areas of the WS shelf [7,76,80]. The subsequent population expansion may have occurred rather recently (approx. 4.25 kyr BP) compared to other regions in which the expansion process occurred much earlier (e.g. EA and AP).

### 4.4. Colonization of the islands

The genetic signal from samples collected from BI and SSI (AI in figure 1) shows a different pattern, reflecting more recent colonization directly from the continent. None of the six haplotypes from the AI are shared among the two island populations. The haplotype network shows the three haplotypes from BI closely related to WS (haplotypes 30, 31, 36; figure 2), while the three haplotypes from SSI are closer to EA (haplotypes 1, 5, 37; figure 2). This result is also well supported by the Bayesian-based clustering analysis that segregated each island into two distinct groups (figure 4). The configuration of the AI haplotypes within the network suggests that BI haplotypes are more divergent from WS, than the SSI ones from EA. This could be explained by the remoteness of BI, located at approximately 1700 km from the continent or any other island [81]. It might be feasible that a small number of migrants from the WS (H31) colonized the isolated island aided by the Weddell Gyre [81] and later derived into H30 and H36 (haplotypes exclusive to BI). In the sea spider *C. megalonyx* a strong connection between populations from BI and WS is also proposed, but in contrast to *N. australe*, *C. megalonyx* from BI (*n* = 43) belong to a single haplotype that is also the most common in the SSI [16]. The *N. australe* results support the notion of a recent, ‘founder effect’ type of colonization of BI from the WS, rather than a potential insular refugium, a hypothesis already proposed for a crinoid species [52]. This scenario would agree with other genetic evidence, suggesting that ocean currents transport adults and larvae from Antarctic shelf areas to the outlying islands, rather than vice versa [7,16,27,77]. Larger sample sizes and better cover of neighbouring Antarctic continental shores are needed to better understand these colonization processes.

Haplotype sharing between the volcanic SSI and the EA region is somewhat unexpected for *N. australe*. Effect of homoplasy in an expanding population is a possibility, but also animals being transported from EA by oceanic hitchhiking or rafting, or by drifting on the ACC has been proposed [82]. SSI faunal composition has been shown as one of the most dissimilar when compared with the Antarctic continental shelf [7,20]; seemingly, the ‘stop’ at SSI might act as a filter retaining individuals, as suggested by the benthic insular refuge hypothesis [20,81]. In *N. australe*, this event seems relatively recent as haplotypes from SSI are mostly shared with EA haplotypes (figure 2). A similar pattern was found for the Antarctic octopus *Paraledone turqueti* [9].

The low genetic diversity together with indication of strong and significant population growth in *N. australe* from the AI may suggest expansion processes after the colonization from the continent, i.e. the expansion after the founder effect. Further sampling of *N. australe* from sub-AI and Antarctic archipelagos is needed for a better understanding of population patterns.

### 4.5. Genetic structure within the East Antarctic

The *N. australe* EA populations were represented by 50 distinct haplotypes leading to relative considerable levels of genetic diversity (table 1). The most probable ancestral haplotype (H5), and the star-like shape network (figure 2*b*) reveal a lack of geographical structure within the EA. Low *F*_ST_ estimates among localities and Bayesian clustering corroborated a single group (i.e. BR, TA and RS localities), except for the DS samples. In contrast to the AP with its complex topography seemingly facilitating the heterogeneity of populations within the region [9,27], the EA populations appear as short branches of the network, suggesting either gene flow with small differentiation among localities or very recent patterns of divergence. Although the near shore EA area is complex with small rocky islands and fjords, depths increase rapidly to greater than 200 m where most of our samples were collected, reaching open basins of sedimentary substrate [83]. It is likely that such conditions are not necessarily barriers to gene flow and could lead to connectivity in organisms with a high bathymetric plasticity [82] as is the case of *N. australe*. On the contrary, species restricted to shallow waters might form distinct faunistic communities on rocks and algae [83] and may find more difficulty in circulating even short distances and thus presenting high levels of genetic substructure as observed in the amphipod *Orchomenella* [59]. The slight differences between the RS and the BR and TA populations might suggest either a glacial refugium in the RS coastal ice-free polynyas [78] or movement of TA individuals to the RS polynyas during periods of ice sheet coverage.

Pycnogonids from DS seem to represent the most genetically distinct population. DS is enclosed in a well-sheltered ice-free bay of 400 km^2^ situated in the Vestfold Hills (see www.antarctica.gov.au). Segregation may be attributed either to a depth constriction (a maximum depth of 25 m) or to geographical isolation. This genetic differentiation of the DS samples from the remaining EA individuals is also found in other studies [52,58] and may be attributable to its local geographical and oceanographic conditions, given the extensive shallow areas of the Vestfold Hills coastal region trapping the shallow fauna forcing them to be inshore residents. Haplotype diversity in the DS population is rather low compared to the remaining EA areas, 10 out of the 13 individuals had the same haplotype H58 (figure 2*b*), probably because all samples were taken from a relatively small area by SCUBA.

Clearly, *N. australe* genetic structure within EA suggest that the eurybathic condition of the species has allowed gene flow over extensive areas of EA, as there is no evidence of bathymetric constraint for the migration of this species. Individuals collected by the REVOLTA program from TA at 40 m depth clustered with deeper waters samples from EA collected between 200 and 1230 m.

In conclusion, our results confirm that *N. australe* is one of the Antarctic brooding invertebrates with the widest distribution. The species shows different demographic histories depending on the Antarctic region, possibly shaped by the characteristics of the deglaciation events post-LGM. The study reinforces the notion of the strong effect of climatic events and environmental conditions on the patterns of diversity and structure in Antarctic benthic fauna. *Nymphon australe* is a key species for the understanding of microevolutionary forces that could reveal connectivity and dispersal mechanisms and demographic processes in the SO.

## Supplementary Material

Electronic Supplementary material S1 Sample location

## Supplementary Material

Electronic Supplementary material S2 Regions and haplotypes

## Supplementary Material

Electronic Supplementary material S3 Genebank accession numbers

## Supplementary Material

Electronic Supplementary material S4 AMOVA results

## Supplementary Material

Electronic Supplementary material S5 Observed and expected mismatch distributions of pairwise haplotype differences

## Data accessibility

The sequences generated in this study have been deposited in GenBank, accession numbers are available through the electronic supplementary material. The whole dataset (sequences file and individuals location data) created within this study has been uploaded to the Australian Antarctic Data Centre https://data.aad.gov.au/.
