# Supplementary figures and images for: Genetic signature of Last Glacial Maximum regional refugia in a circum-Antarctic sea spider
Source: R Soc Open Sci. 2017 Oct 18;4(10):170615. doi: 10.1098/rsos.170615 (PMC5666255; doi:10.1098/rsos.170615)

A

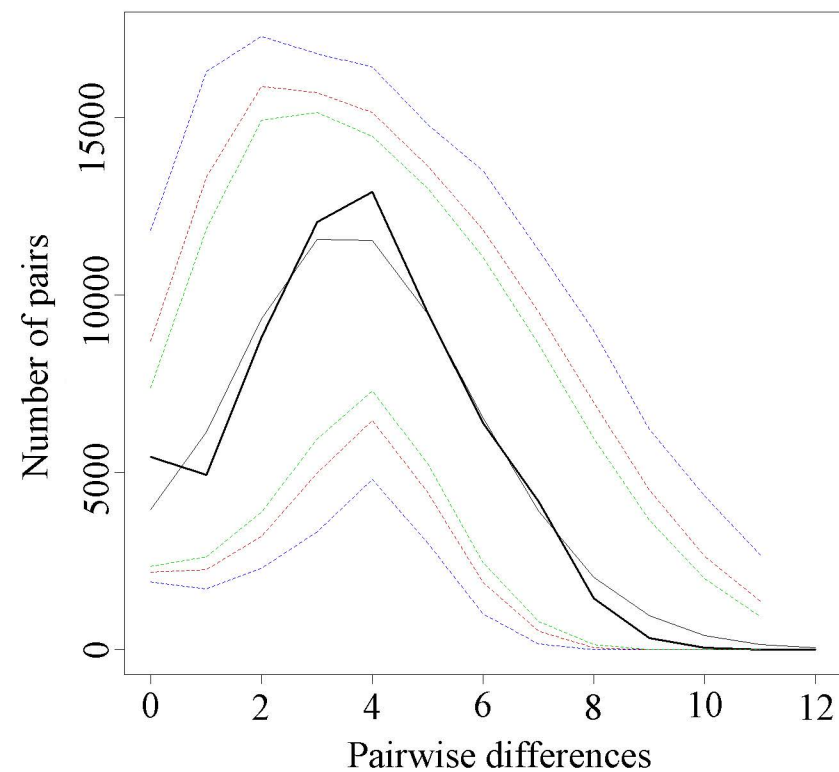

B

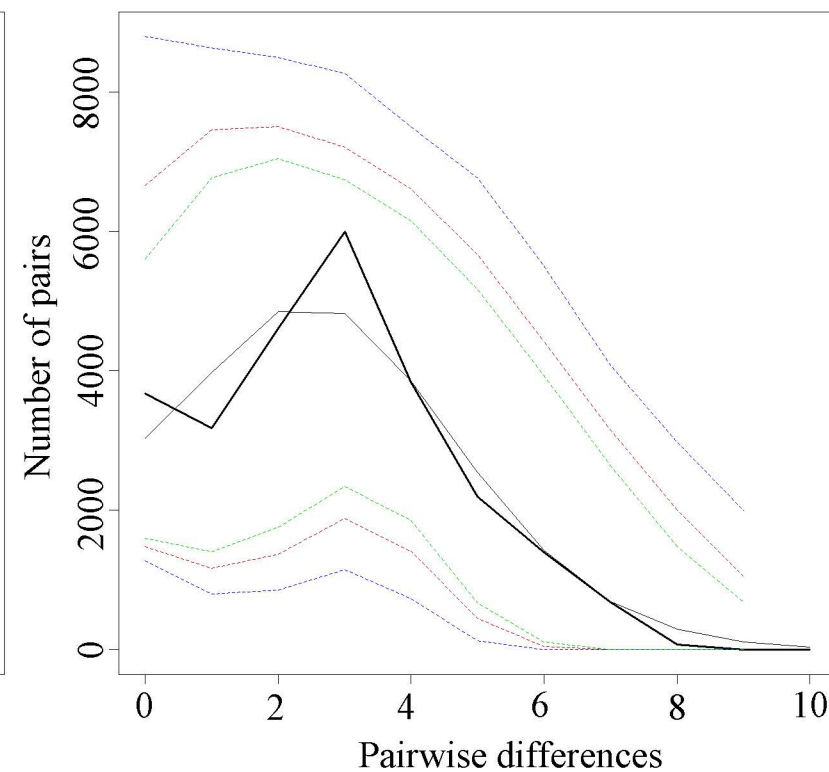

C

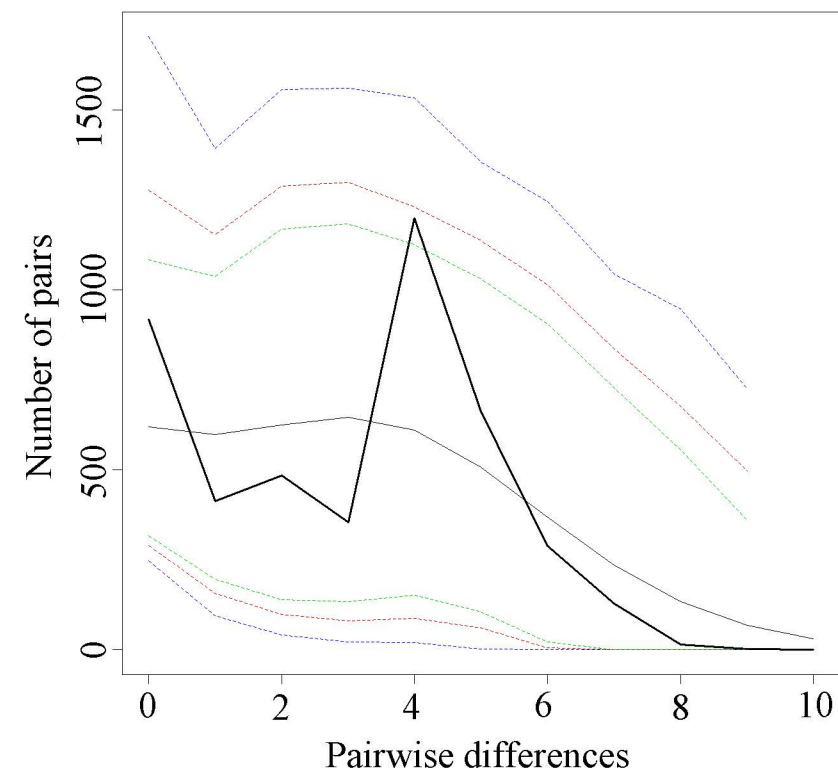

D

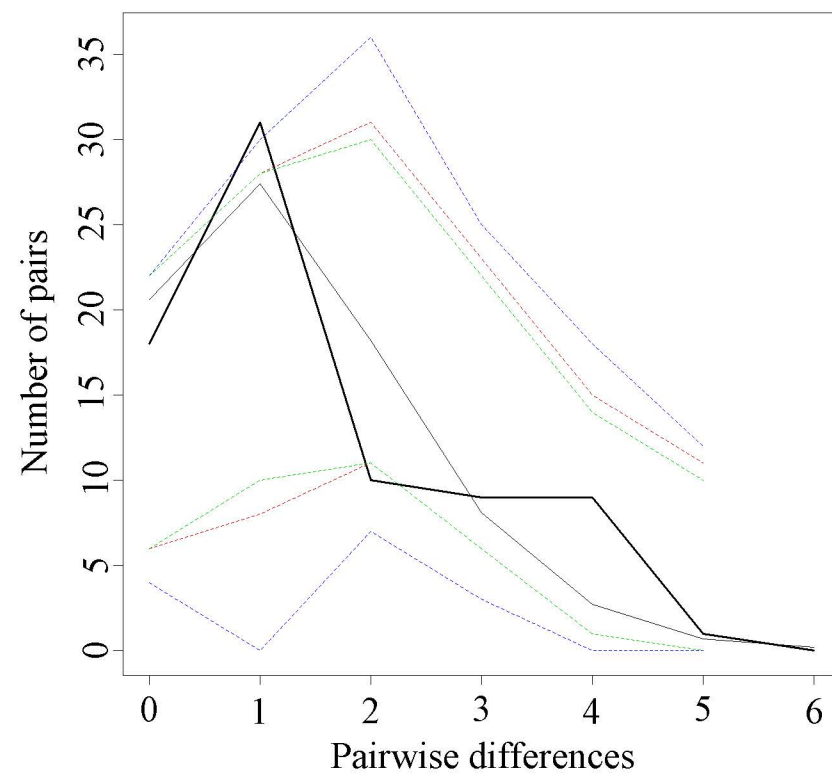

E

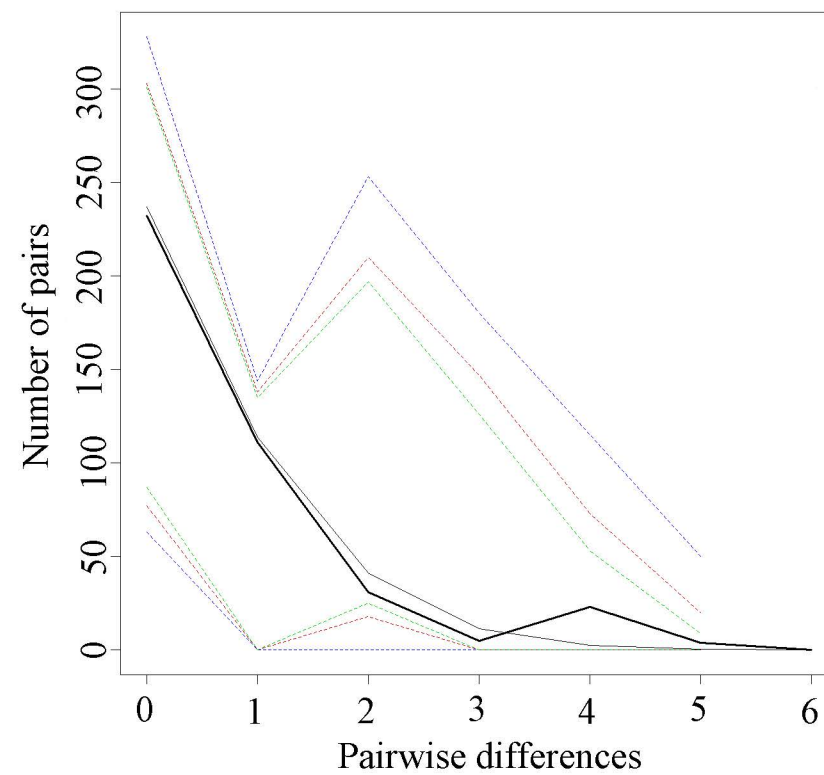

— observed  
 — expected  
 - - - 99% CI  
 - - - 95% CI  
 - - - 90% CI

Supplement: Electronic Supplementary material S5 Observed and expected mismatch distributions of pairwise haplotype differences [file rsos170615supp5.pdf]
